# Functionality and Scar Evaluation of the Donor Site in Extended Radial Forearm Flap Phalloplasty: How Affected Is the Arm in Day-to-Day Life? †

**DOI:** 10.3390/jcm13196004

**Published:** 2024-10-09

**Authors:** Mahmut Ozturk, Sascha Wellenbrock, Philipp Wiebringhaus, Marie-Luise Aitzetmüller-Klietz, Lara Küenzlen, Anna Burger, Sahra Nasim, Tobias Hirsch, Matthias Aitzetmüller-Klietz, Baksan Tav, Ulrich M. Rieger

**Affiliations:** 1 Department of Plastic Surgery, Center for Transgender Health, University Hospital Muenster, 48149 Muenster, Germanyphilippwie@gmx.de (P.W.); marie.luise.klietz@gmx.de (M.-L.A.-K.); aitzetmueller.m@hotmail.com (M.A.-K.); baksan@gmail.com (B.T.); 2 Department of Plastic and Reconstructive Surgery, Institute for Musculoskeletal Medicine, 48143 Muenster, Germany; 3 Department of Plastic, Reconstructive and Aesthetic Surgery, Hand Surgery, Fachklinik Hornheide, 48157 Muenster, Germany; 4 Department of Plastic & Aesthetic, Reconstructive & Hand Surgery, AGAPLESION Markus Hospital, Academic Teaching Hospital of the Goethe University, 60431 Frankfurt am Main, Germany; l.kasper@live.de (L.K.); ulrich.rieger@agaplesion.de (U.M.R.); 5 Department of Plastic Surgery, University Hospital Zurich, Rämistr. 100, 8091 Zurich, Switzerland; anna.burger@usz.ch; 6 Department of Plastic Surgery, Alfred Krupp Hospital, Hellweg 100, 45276 Essen, Germany; sahranasim@outlook.de

**Keywords:** genital masculinization, radial forearm free flap, gender-affirming surgery, phalloplasty, donor site morbidity

## Abstract

**Background:** The radial forearm flap remains the gold standard in phalloplasty in gender-affirming surgery due to its versatility and functional outcome, but the significant donor site morbidity and its impact on daily functioning and aesthetic perception remains understudied. This study provides valuable insights into the mid-term functional and aesthetic outcomes of the forearm in transgender individuals following radial forearm flap phalloplasty using widespread instruments for assessment scoring systems and for the evaluation of postoperative wound healing of surgical interventions in general. **Methods:** Between January 2013 and March 2018, a total of 47 patients underwent radial phalloplasty at AGAPLESION Markus Hospital, and 20 consented to participate in this cross-sectional, retrospective study evaluating functional and aesthetic outcomes post-radial forearm flap phalloplasty using standardized questionnaires (DASH, POSAS, and SBSES). A univariate median regression of each score was performed to determine the associations with selected variables, and correlation analyses between scores was performed using a nonparametric Spearman rank correlation. **Results:** Among the 20 participants, the median Quick DASH, DASH functionality, DASH sport and music, and DASH work scores indicated minimal to no functional impairment. Scar evaluations using the PSAS, OSAS, and SBSES scales showed overall patient satisfaction with minimal concerns regarding pigmentation and scar texture. Notably, an increase of 0.27 cm^2^ in wound surface area added one point to the DASH sport and music score (*p* = 0.037). Statistical analysis also demonstrated a significant correlation between functional and aesthetic assessment scores. **Conclusions:** The radial forearm phalloplasty donor site, evaluated by the PSAS, OSAS, and SBSES scales, leads to minimal or no functional impairment; however, the worsening of the DASH sport and music score with increasing wound surface reflects a direct relation between wound size and functional impairment.

## 1. Introduction

The term “phalloplasty” was initially introduced by Spengler in 1858 to delineate the reconstruction of a penis following degloving injury. Today, phalloplasty is primarily recognized as the third step in gender-affirming surgery, succeeding the subcutaneous mastectomy, and preceding the hysterectomy and bilateral oophorectomy [1].

In penile reconstruction, the ideal outcome should be achieved in a single procedure, yielding an aesthetically acceptable result that retains both erogenous and tactile sensation, enables urination while standing, and allows for penetrative sexual intercourse [2,3]. Among the available options, such as the osteocutaneous fibula, musculocutaneous latissimus or gracilis flaps, and fasciocutaneous thigh flaps, the free radial forearm flap (RFFF) is preferred. Its advantages include superior pliability, a reliable large-caliber artery, and the ability to integrate foreign materials such as penile prostheses, making it the closest approximation for the desired, though still challenging, reconstruction.

A significant milestone in RFFF phalloplasty was achieved with Chang and Hwang’s introduction of the tube-in-tube concept [4]. This innovative technique facilitated the incorporation of the urethra within a free flap penile-like framework within a single-stage procedure. Despite the existence of alternative flap options, the one-staged RFFF phalloplasty remains the gold standard in genital gender affirmation surgery. However, it is crucial to acknowledge the considerable donor site morbidity subsequent to the RFFF phalloplasty, affecting nearly the entire forearm circumference.

Particularly among young transgender men, there exists a significant concern pertaining to potential functional impairments and exposure to aesthetic stigmata, which could hinder their ability “to blend in” with society. The assessment of upper extremity performance and the aesthetic implications associated with donor site morbidity has been understudied. Contemporary literature primarily focuses on the evaluation of the range-of-motion of the affected extremity rather than elaborating the broader impact of the surgery on daily functioning.

Hence, this study aims to analyze the functional as well as aesthetic outcome after RFFF phalloplasty in transgender individuals using standardized questionnaires.

A reliable tool for evaluating functional impairment in the donor forearm is the Disabilities of the Arm, Shoulder, and Hand (DASH) questionnaire. It is specifically designed to assess upper limb function and related disabilities, making it an ideal tool. Since the radial forearm free flap involves significant soft tissue manipulation and potential long-term changes in dexterity, grip strength, and overall arm function, DASH provides a comprehensive assessment of how the donor site impacts daily activities and quality of life.

Scar evaluation tools, namely the Patient and Observer Scar Assessment Scale (POSAS) and the Stony Brook Scar Evaluation Scale (SBSES), are validated tools for assessing scar quality. In the POSAS, both the patient’s and the clinician’s perspectives are assessed, while the SBSES focuses mainly on linear scars, such as those seen at the donor site following radial forearm phalloplasty. Donor site scars are often large and may have both functional and aesthetic consequences. Therefore, the POSAS questionnaire captures subjective patient experiences, such as pain, itching, and scar appearance, as well as objective clinical evaluations regarding pigmentation, texture, and overall scar condition, whereas the SBSES provides a rapid, standardized, and objective method for assessing scar appearance, offering clear criteria for successful wound healing and cosmetic outcomes [5,6]. The inclusion of both patient and observer evaluations provides a more nuanced and holistic assessment of scar outcomes compared to other scar evaluation scales.

Due to their established validity, reliability, and ability to provide comprehensive assessments of both functional outcomes and scar-related morbidity at the donor site, these specific instruments were selected over others. Together, they offer a balanced and multidimensional evaluation, addressing both functional impairments and the aesthetic and sensory consequences of the radial forearm flap procedure.

## 2. Materials and Methods

### 2.1. Ethical Statement

The study was approved by the Ethics Committee of the Hessian State Chamber of Physicians (Number FF25/2017).

### 2.2. Study Design and Sample Composition

A cross-sectional retrospective analysis was performed using standardized DASH, POSAS, and SBSES questionnaires on patients who underwent gender-affirming radial forearm flap phalloplasty at the Department for Plastic and Aesthetic Surgery, Reconstructive and Hand Surgery at the AGAPLESION Markus Hospital in Frankfurt am Main, Germany between January 2013 and March 2018. A total of 47 patients were operated on during the study period and were contacted from March 2017 to November 2018. Of these, 20 consented to adhere to the study. Collection of patient characteristics, clinical features, and analysis of the collected data from questionnaires were performed retrospectively.

### 2.3. Surgical Procedure

The surgical procedure was standardized utilizing the technique described by Gottlieb and Levine to prevent any technical-related bias [7]. Preoperatively, Allen’s test was performed in all patients, and only those that showed normal test results underwent surgery.

The flap design, Allen test, and sonographic surveillance of the course of the radial and recipient deep inferior epigastric arteries, as well as the proximal greater saphenous vein, were performed prior to surgery. All surgical procedures were carried out by two senior doctors with one or two residents in a two-team approach under general anesthesia in a lithotomy position with the left arm placed on an arm table.

**Figure 1:**
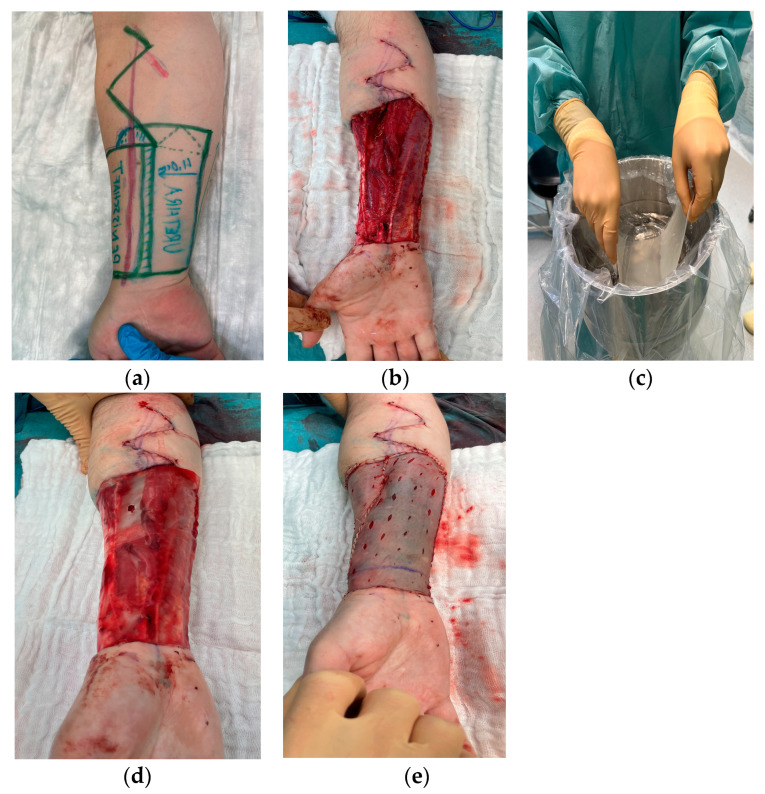
(**a**–**e**) Case example of a radial phalloplasty in a transmasculine patient. Preoperative planning and preparation of the forearm flap; extensive donor site surface with partially exposed tendons after flap elevation; preparation of the collagen sheet graft; post inset of the graft with the sutured split skin graft of the lateral thigh in place.

Typically, the flap harvested for reconstruction encompasses the entire palmar and a significant portion of the dorsoradial side of the non-dominant forearm. Firstly, the direct Allen test was performed with a micro clamp on the radial artery. Then flap elevation continued under an inflated tourniquet to 260 mmHg along the surgical markings from the lateral to medial direction towards the neourethra. A strip of 1 cm on each side was de-epithelialized. The radial artery was clipped distally with its venae commitantes, and the dissection proceeded proximally. Towards the cubital fossa, the lateral and posterior antebrachial cutaneous nerves as well as the cephalic vein were dissected, and the radial artery was identified and clipped right before disemboguing in the brachial artery. Afterwards, the urethral catheter was placed in position and the neophallus was wrapped around the catheter, as well as itself, and was sutured with PDS 5 × 0. The flap anastomosis on the recipient site was performed while suturing the wound edges on the proximal forearm together. A split skin graft (0.2 mm thickness) was harvested from the lateral thigh via a dermatome to cover the palmar and dorsal wound surface with a sheet of collagen matrix below. Alternatively, a full thickness skin graft from the groin was used and a vacuum-assisted closure with 125 mmHg negative pressure was applied to complete the donor site surgery (Figure 1a–e).

### 2.4. Applied Questionnaires

All participants were in good health and completed the following questionnaires.

#### 2.4.1. Functionality Evaluation

##### The Disabilities of the Arm, Shoulder, and Hand (DASH)

The Disabilities of the Arm, Shoulder, and Hand questionnaire measures impairments and activities of daily living after single or multiple disorders of the upper limb [8]. Besides the 30 item self-reported questionnaire, there were two additional sections focusing on sports/music and work outcomes.

The response options ranged from “no difficulty” with a score of 1, to “unable” with a score of 5. The sum of the 30 items led to a DASH score ranging from 0 to 100. Higher scores indicated more lower arm impairment.

#### 2.4.2. Scar Evaluation

##### Patient and Observer Scar Assessment Scale (POSAS)

The Patient and Observer Scar Assessment Scale is a standardized questionnaire obtaining a doctor’s as well as a patient’s own evaluation of the donor site scarring.

The observer’s assessment contained five items: the degree of scar vascularization, pigmentation, thickness, relief, and pliability. The patient’s evaluation includes six items: the degree of pain, itching, and whether the scar was firm, irregular, and showed differences in thickness or color. Every item was scored from a score of 1, denoting “normal skin”, to 10, denoting “worst imaginable scar or sensation”. The total score of the OSAS resulted from the addition of the scores for five items and ranged from 5 to 50. Similarly, the PSAS included the scar evaluation of an observer, and a higher PSAS score indicated a worse scarring evaluation.

Various studies have shown the reliability of the POSAS for analyzing the quality of a scar by combining patient and observer assessments [9,10,11,12].

##### The Stony Brook Scar Evaluation Scale (SBSES)

The Stony Brook Scar Evaluation Scale rates five items on a dichotomous ordinal scale. It incorporates individual attributes analyzed by the observer in a binary response (0 or 1) for each field. The sum of all the fields range from 0, “worst”, to 5, “best scar/normal skin”. Higher scores indicate better evaluation scores. The SBSES has been acknowledged as a valuable tool in assessing the long-term aesthetic appearance of scars [13].

### 2.5. Statistical Analysis

Continuous data are described using medians, interquartile ranges (IQR), and ranges, and categorical data are presented as frequencies and percentages. The normality of continuous data was evaluated using the Shapiro–Wilk test. The univariate median regression of each score was performed to determine associations with selected variables. Results of the median regression are expressed as coefficients with a 95% confidence interval (CI) and *p* value. Correlation analyses between scores were performed using the nonparametric Spearman rank correlation with a corresponding *p* value. All statistical analyses were performed using Stata software (version 17, Stata Corp LLC, College Station, TX, USA). A two-tailed *p* < 0.05 was used to determine statistical significance.

## 3. Results 

Between January 2013 and March 2018, a total of 47 gender-affirming phalloplasty procedures were performed at our department. Among those, 20 patients adhered to the study. All included patients received the tube-in-tube technique described by Gottlieb and Levine [7]. The average duration of the procedure was 499 min (467, 581 min). The median follow-up time was 39 (33, 51) months. Median body mass index (BMI) at the time of procedure was 24,8 kg/m^2^ (23.3, 26.8 kg/m^2^). The median age at the time of the surgery was 35 (22, 42). The median penile length was 11 cm (10, 12.3 cm). In 12 (60%) patients, the radial flap was harvested from the left forearm. The median wound surface was 153.6 cm^2^ (141.5, 172.5 cm^2^). Of all the patients, 15 (75%) underwent donor site coverage on their non-dominant forearm with a full thickness skin graft, and 5 (25%) with Matriderm^®^ (Medskin Solutions Dr. Suwelack AG, Billerbeck, Germany) plus a split skin graft.

Noticeably, tobacco use or former nicotine abuse was present in eleven patients (55%), ranging from 6 to 15 pack-years. Other comorbidities relevant for surgery were two (10%) cases of diabetes mellitus type II and one (5%) case of hypothyroidism with rheumatoid arthritis as a secondary diagnosis.

Two (10%) of the patients reported having chronic pain on the forearm due to scarring. Four patients (20%) required reoperation due to donor site complications: two (10%) due to hematoma on the proximal forearm, one (5%) due to skin necrosis with re-covering with a skin graft, and one (5%) due to a contracted scar that was addressed with lipofilling. 

### 3.1. Functional Outcome

The median Quick DASH, DASH functionality, DASH sport and music, and DASH work scores were 7.1 (1.6, 20.4), 7.1 (1.7, 20.8), 12.5 (0, 18.8), and 9.4 (0, 43.8), respectively (Table 1).

### 3.2. Scar Outcome

#### 3.2.1. PSAS and OSAS

Scores based on patients’ self-assessment (PSAS, with a denominator of 60) were only above the third quartile value (23.5/60) for five (20%) out of the total patients.

**Figure 2:**
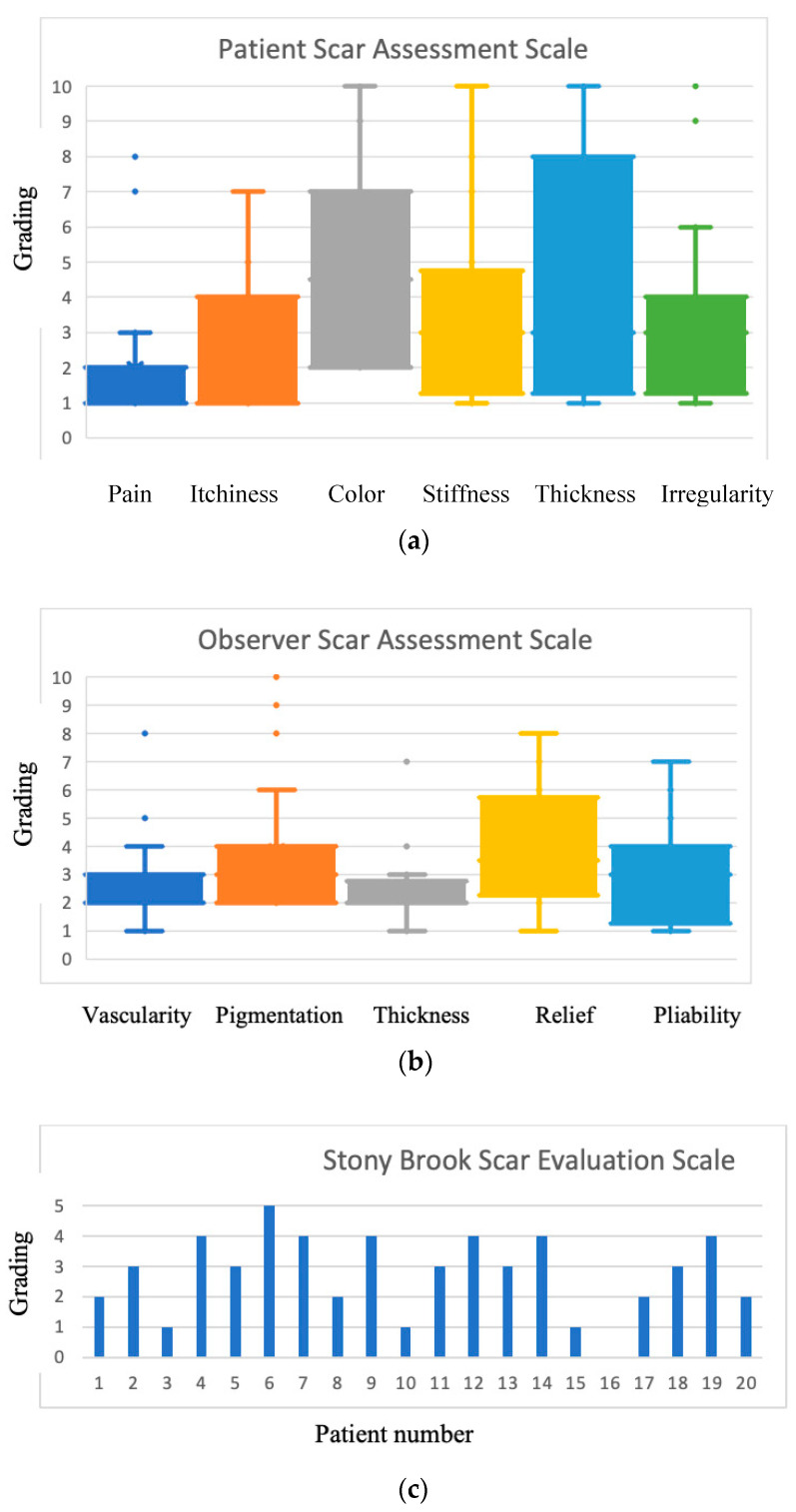
(**a**–**c**) Median overall scores for each evaluation scale.

Similarly, in the OSAS (with a denominator of 50), observers evaluated the donor site in only 5 (20%) patients to be above the third quartile value; however, only two of these patients were overlapping. In PSAS, satisfactory scores for pain and itchiness were observed (median score for each: 1). The OSAS also revealed acceptable scores for vascularization and thickness of the scar (median score for each: 2), whereas the thickness of the scar was scored by patients with a median of 3 out of 10. The median pliability score was 3 in both scales. The median pigmentation score was a slightly higher value of 3 for patients and 3.5 according to observers. The appearance of the scar with the item “color” had the highest median score of all items, with 4.5 out of 10 (Figure 2a,b).

The median overall PSAS and OSAS scores were 18.5 (13.25, 22.5) and 14 (12, 17.25), respectively (Table 1).

#### 3.2.2. The Stony Brook Scar Evaluation Scale

For the SBSES, the median total score was 3 out of 5 (range: 2–4), with seven (35%) patients scoring two or less, indicating an insufficient aesthetic outcome (Figure 2c).

### 3.3. Correlation Analysis

#### Functionality and Wound Surface

**Figure 3:**
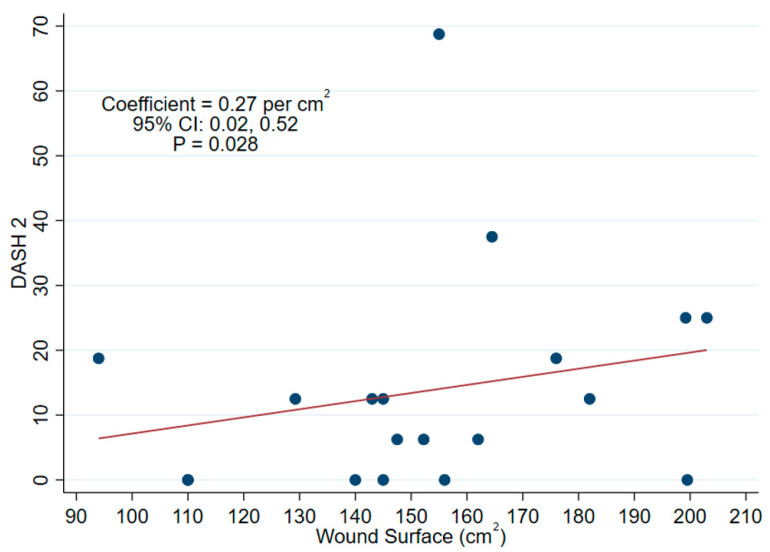
The correlation analysis between the wound surface and Disabilities of the Arm, Shoulder, and Hand for sports and music (DASH 2) scale. An increase of 0.27 cm^2^ in the wound surface worsened the DASH sports and music score by one point.

Univariate median regression analysis, as shown in Table 2 below, revealed a significant correlation between increasing wound surface and the DASH sport and music scores. For each 0.27 cm^2^ increase in the wound surface, the DASH score would increase by one point in this category (Figure 3).

The same analysis did not result in a significant correlation between variables such as age at the time of surgery, age above or below 30, defect closure, penile length, or the observation scores.

### 3.4. Functional Assessment and Scar Evaluation Scores

**Figure 4:**
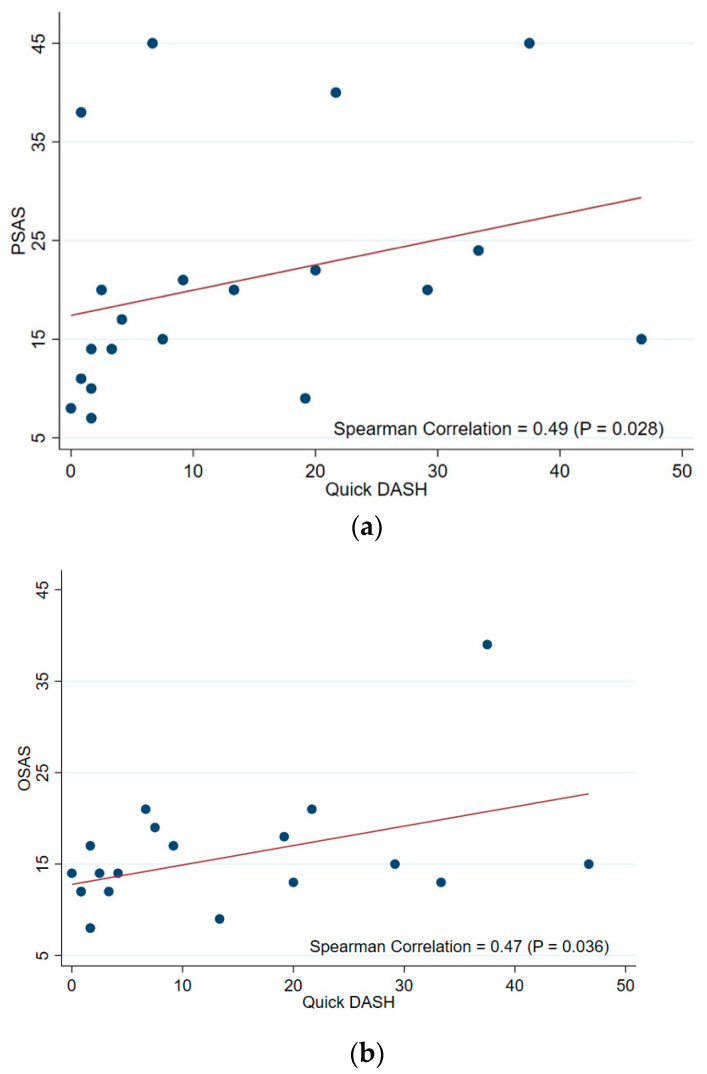
(**a**,**b**) The correlation analysis between (**a**) the Patient Scar Assessment Scale (PSAS) and the Quick Disabilities of the Arm, Shoulder, and Hand (DASH) score (Spearman correlation coefficient of 0.49, *p* = 0.028); and the (**b**) Observer Scar Assessment Scale (OSAS) and Quick Disabilities of the Arm, Shoulder, and Hand (DASH) score (Spearman correlation coefficient of 0.47, *p* = 0.036).

**Figure 5:**
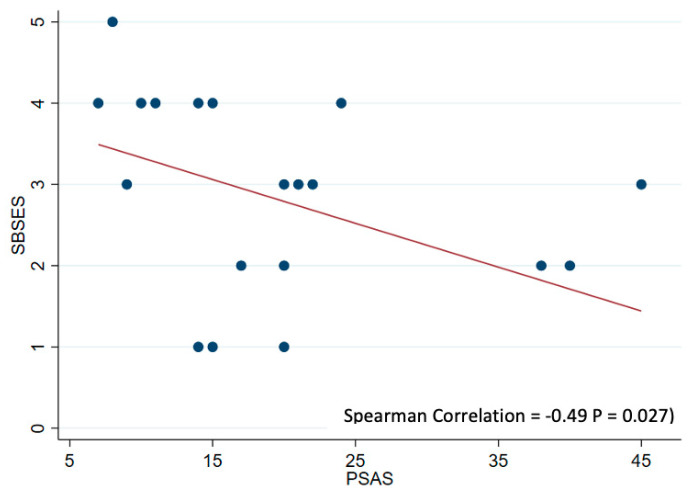
The correlation analysis between the Stony Brook Scar Evaluation Scale (SBSES) and Patient Scar Assessment Scale (PSAS) (Spearman correlation coefficient of −0.49, *p* = 0.027).

**Figure 6:**
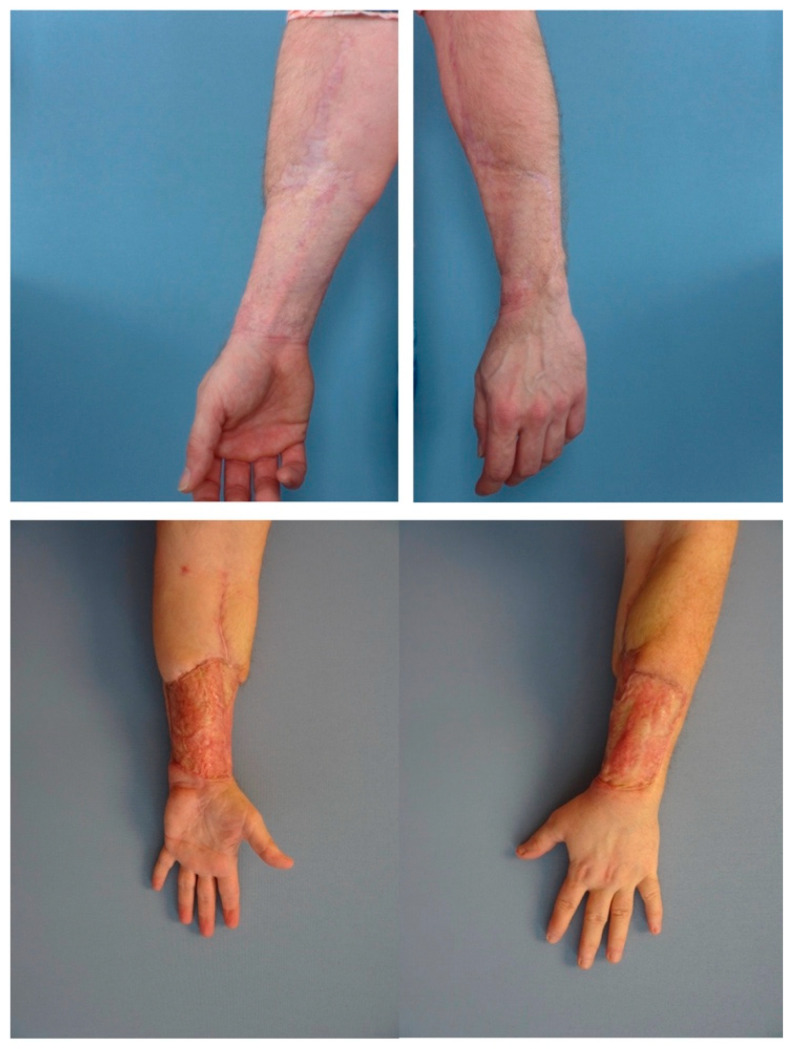
Aesthetic outcome of scar quality in the highest vs. lowest SBSES/POSAS score.

In Table 3, the correlation analysis demonstrates a moderately significant correlation between the Quick DASH score and PSAS (Spearman correlation coefficient of 0.49, *p* = 0.028), as well as the correlation between the Quick DASH score and OSAS (0.47, *p* = 0.036) (Figure 4a,b). A significant negative correlation between the PSAS and SBSES scores (−0.49, *p* = 0.027) was also observed (Figure 5). The substantial difference between the highest and lowest SBSES/POSAS in donor-site scar outcomes is shown in Figure 6.

## 4. Discussion

The RFFF remains the most commonly used technique for phalloplasty due to its versatility, despite extensive donor site morbidity [2,14]. This study, with a median follow-up of over three years, provides valuable insights into the mid-term functional and aesthetic outcomes of the forearm in transgender individuals following RFFF phalloplasty, using scoring systems commonly employed in orthopedic and trauma patients as well as in the evaluation of postoperative wound healing of surgical interventions in general [5,13,15].

Previous studies have reported a range of donor site complications, with Morrison et al. noting a 16% re-grafting rate and 3% scarring incidence [16]. In our sample, reoperations due to re-grafting need, as well as hematoma and aesthetic issues, were less frequent. Patient demographics, including BMI, age, and tobacco use, were comparable to other studies [17,18].

The univariate median regression and correlation analyses identified a correlation between an increasing wound surface and the DASH sport and music scores. Enlarging the wound surface by 0.27 cm^2^ worsened the DASH score in sport and music significantly. This finding confirmed the hypothesis of Salvaggi et al. that the donor site morbidity increased proportionally to the dimensions of the flap, emphasizing the importance of considering both functional and aesthetic aspects as well as tailored patient communication in surgical planning [2]. Thorough discussions on flap size and potential postoperative disability with patients can align expectations with medical necessities and mitigate patient dissatisfaction. In this cohort, all patients were operated on with the Gottlieb and Levine technique [7], achieving an average length of 11.3 cm and a width of 13.6 cm with a mean flap size of 154.1 cm^2^, which was smaller than in comparative studies that ranged from 7.5 to 14 cm in length, a width up to 17 cm, and an average donor site area of 228 cm^2^ [19,20,21].

### 4.1. Functional Outcome Analysis

Clinically, one would expect that post-radial forearm flap patients undergoing genital masculinization experience some degree of functional impairment due to extensive tissue harvesting. However, our findings indicate that the median DASH scores, including the Quick DASH, DASH functionality, DASH sport and music, and DASH work scores, suggest minimal or no functional impairment in the majority of patients, and thus, does not support this initial hypothesis. These results align with previous studies that have demonstrated similar outcomes in terms of grip strength and nerve function post-phalloplasty [21]. Notably, the DASH scores in our cohort did not differ significantly from those reported in age-matched healthy populations, supporting the conclusion that RFFF phalloplasty does not result in significant functional impairment. This suggests that our surgical technique effectively preserved upper limb mobility. However, a relevant limitation is the negative correlation between the size of the wound surface and the DASH score, indicating that larger wound areas may still contribute to some degree of functional impairment.

Therefore, candidate selection should involve careful evaluation of forearm size and tissue quality, as individuals with smaller forearms or thinner skin may be at a higher risk for functional limitations post-surgery. In such cases, alternative donor sites (e.g., the anterolateral thigh) could be considered to preserve function while still achieving the desired aesthetic and reconstructive goals.

Watfa et al. utilized the DASH questionnaire to assess the outcome after full thickness skin grafting (FTSG) and Matriderm plus split skin grafting and demonstrated a significant difference between those groups, showing a mean DASH score of 15.7 for patients with FTSG versus 0.43 in those with Matriderm [22]. A similar analysis between these two groups did not yield a significant difference in any of the scores that were utilized in our study.

### 4.2. Scar Quality Assessment

The median scores derived from the PSAS, OSAS, and SBSES assessments collectively demonstrated overall patient satisfaction regarding aesthetic outcomes.

Only in two cases did both patient and observer scores overlap on a relatively unfavorable outcome. The utilized scar evaluation scores yielded moderately average results overall; however, the observed impairment in color and thickness of the scar, as well as the relief, elucidated the fact that there is still room for improvement of the donor site scar quality, which could potentially minimize stigmatization (Figure 1a,b). The limited concurrence in evaluations suggests possible disparities in perception between patients and external observers, underscoring the necessity for comprehensive assessment tools. For transgender patients, scars are an additional emotional weight, where the current patient score might indicate dissatisfaction not only with their physical appearance but also with the scar’s impact on their gender affirmation process.

Current literature underlines the significant impact of visible scarring on mental health outcomes, as it can exacerbate gender dysphoria, affect self-esteem, and influence social interactions [23,24]. Clinicians should interpret patient-reported scores in this context, recognizing that even clinically “acceptable” scars may cause psychological distress in transgender individuals.

The discrepancy in scar evaluations by observers underscores the need for the normalization of such scars for society.

From a surgical perspective, the method of dissection could potentially impact postoperative angiogenesis as well as graft take, as both play a major role in postoperative scar quality. Shonka et al. suggested that suprafascial harvesting would decrease the risk of tendon exposure. Similarly anatomic as well as retrospective studies also advised using suprafascial harvest of the radial flap for head and neck reconstruction [25,26,27]. In our patient group, we exclusively performed suprafacial harvesting and did not face any major complications, such as tendon exposure. Considering the negative impact of incomplete graft take or re-grafting on aesthetic outcome [2], the utmost importance was given to proper flap harvesting and split skin grafting.

The shift in defect closure from full thickness skin to Matriderm with split skin (STSG) in our practice could also have possibly played a role in reduced hyperpigmentation, as well as on the smoother texture of the graft, most probably due to the larger diffusion distance in FTSG [28].

In their study with a follow-up period of seven years, Caenegem et al. performed a questionnaire-based analysis using the POSAS with 44 transgender patients asking for masculinization bottom surgery and reported worse scores for stiffness and thickness (5/10) and that both groups rated a more pliable scar later in follow-up [19]. Based on the fact that patient follow-up ranged from two to five years in our study, we could expect to have even better scar evaluation scores in a future follow-up study on the same patients.

It is important to highlight in the literature that transgender patients differ from other groups of patients because of the pre-existing burden of gender dysphoria. While the completion of the surgery using a penile prosthesis may come with opportunities for standing urination, erectile function, and, perhaps, sexual satisfaction, the resultant scarring of the donor site on the forearm can be another focused issue for these patients. Such scarring can be detrimental to quality of life and can add another dimension to the overall well-being of a person [2,3].

Careful patient selection is needed, especially when there are high concerns for visible scarring, and those with pre-existing body image issues may benefit from detailed preoperative counseling about the potential for donor site scarring and its impact on their overall well-being.

### 4.3. Correlation Analysis Functional and Aesthetic Assessment Tools

Cross-checking of the utilized scores in our cohort also demonstrated significant implications regarding the surveillance of functional and aesthetic outcomes following radial phalloplasty. The findings indicated a significant correlation between QuickDASH and PSAS scores, QuickDASH and OSAS scores, and PSAS and SBSES scores in assessing postoperative arm function in these patients. These results do, in fact, show that patients return consistent results for each of the proposed tests, regardless of the test used. The significant correlations among the tests further confirm their potential or, at least, the potential of one or more tests used in the present study to be introduced into future standardized clinical follow-ups of the same or similar patient populations. One might also consider this as an additional safeguard to ensure the feasibility of incorporating these tests into clinical practice.

## 5. Limitations

This study focuses primarily on the assessment of the upper extremity and functional outcomes, propagating future research for broader implications for daily functioning, quality of life, and mental health.

Especially across all applied scores, general limitations need to be underlined. None of these questionnaires directly assessed the psychological impact of donor site morbidity on transgender individuals, which is of utmost importance, compared to non-transgender populations when asked for their subjective opinion, such as in the POSAS. The absence of cultural sensitivity and the need for gender-specific, inclusive language in this sample must be noted.

Despite exhausting all outreach methods, the limited number of patients is probably due to the long distances that patients were required to travel, as they came from all around the country, and the retrospective nature of the study, which could potentially limit the generalizability.

The limitations of this retrospective evaluation include selection bias, with patients who have already undergone the procedure, and the inability to control for confounding variables, which highlights the need for more robust prospective studies. Future prospective studies could provide a clearer and more accurate understanding of the functional and aesthetic outcomes following radial forearm free flap phalloplasty by conducting randomized controlled trials. This may lead to an algorithm decision-making process regarding donor site selection and surgical techniques, successively improving patient outcomes and satisfaction.

## 6. Conclusions

The findings of this study endorsed the suitability of RFFF phalloplasty for gender-affirming surgery with acceptable postoperative donor site morbidity. The fact, that the scores in the cohort did not significantly deviate from healthy, age-matched populations suggests that, for most patients, the surgery does not result in substantial long-term upper limb impairment. This is particularly reassuring for individuals concerned about the potential loss of hand or arm function, making RFFF a suitable option for a broad range of candidates. Despite the fact that RFFF led to minimal to no functional impairments, each addition of 0.27 cm^2^ in the wound surface size added one point to the DASH score, reflecting a direct relation between wound size and functional impairment.

Stigmatizing of aesthetic scarring concerns, measured by the PSAS, OSAS, and SBSES scales, suggests a need for further refinement in surgical techniques and the significance of patient counseling to lessen the psychosocial consequences of visible scars.

## Figures and Tables

**Table 1:** Patient characteristic and surgical scar assessment data.

Surgical and Scar Assessment Data	
Variable	n (%) or Median (IQR) [Range]
**Number of Patients**	**20**
**Age**	35 (22, 42) [18, 46]
**Age category**	
<30	9 (45%)
>30	11 (55%)
**Weight**	72 (65, 78)
**Height**	167 (164, 168)
**BMI**	24.8 (23.3–26.8)
**Wound surface (cm^2^)**	153.6 (141.5, 172.5) [94, 203]
**Defect closure type**	
Full thickness skin graft	15 (75%)
Matriderm + split skin	5 (25%)
**Graft Length**	11 (10, 12.5) [8, 14]
**DASH Value Part 1 “Functionality”**	7.1 (1.7, 20.8) [0, 46.7]
**DASH Part 2 “Sport und Music”**	12.5 (0, 18.8) [0, 68.8]
**DASH Part 3 “Work”**	9.4 (0, 43.8) [0, 93.8]
**Patient observation (6–60) PSAS**	19 (13, 23) [7, 45]
**Observer score (5–50) OSAS**	14 (12, 18) [8, 39]
**SBSES**	3 (2, 4) [0, 5]

**Table 2:** Results are presented as a coefficient (95% confidence interval), *p* < 0.05 marked as * significant data. Significant results are bolded. DASH: Disabilities of the Arm, Shoulder, and Hand; POSAS: the Patient and Observer Scar Assessment Scale; SBSES: Stony Brook Scar Evaluation Scale.

Univariate Median Regression Analysis of Scores						
Variable	DASH Value Part 1 “Functionality”	DASH Part 2 “Sport und Music”	DASH Part 3 “Work”	Patient Observation (6–60)	Observer Score (5–50)	SBSES
**Age**	0.62 (−0.36, 1.59) *p* = 0.2	0.37 (−0.77, 1.5) *p* = 0.503	1.44 (−0.17, 3.1) *p* = 0.076	0.26 (−0.71, 1.23) *p* = 0.578	0.12 (−0.18, 0.42) *p* = 0.411	0.04 (−0.04, 0.12) *p* = 0.293
**Age > 30**	10 (−9.6, 29.6) *p* = 0.298	6.3 (−11.9, 24.4) *p* = 0.478	12.5 (−13.4, 38.4) *p* = 0.321	5 (−14.6, 24.6) *p* = 0.598	3 (−3.2, 9.2) *p* = 0.32	1 (−0.6, 2.6) *p* = 0.21
**Wound surface (cm^2^)**	−0.01 (−0.38, 0.35) *p* = 0.933	**0.27 (0.02, 0.52)** *p* = 0.037 *	0.1 (−0.75, 0.96) *p* = 0.8	0.08 (−0.3, 0.45) *p* = 0.678	−0.04 (−3.17, 9.17) *p* = 0.587	0 (−0.04, 0.04) *p* = 0.999
**Defect closure type**						
Full thickness skin graft	Reference	Reference	Reference	Reference	Reference	Reference
Matriderm +split skin	−3.3 (−25.9, 19.2) *p* = 0.76	6.3 (−14.2, 26.6) *p* = 0.526	0 (−44.4, 44.4) *p* = 0.999	−6 (−28.7, 16.7) *p* = 0.586	0 (−10.8, 10.8) *p* = 0.999	−1 (−2.9, 0.9) *p* = 0.282
**Length**	−1.7 (−7.2, 3.9) *p* = 0.537	2.1 (−2.8, 7)	2.1 (−9.8, 14) *p* = 0.715	1 (−4.8, 6.8) *p* = 0.722	−0.7 (−2.1, 0.6) *p* = 0.26	0 (−0.62, 0.62) *p* = 0.999
		*p* = 0.38				

**Table 3:** Correlation analysis among evaluation scores. DASH: Disabilities of the Arm, Shoulder, and Hand; POSAS (PSAS and OSAS): Patient and Oberserver Scar Assessment Scale; SBSES: Stony Brook Scar Evaluation Scale. Significant results are bolded; *p* < 0.05 marked as * significant data.

Correlation Analysis		
Comparison	Spearman Correlation	*p* Value
**Quick Dash versus PSAS**	0.49	0.028 *
**Quick Dash versus OSAS**	0.47	0.036 *
**Quick Dash versus SBSES**	−0.27	0.252
**PSAS versus OSAS**	0.43	0.059
**PSAS versus SBSES**	−0.49	0.027 *
**OSAS versus SBSES**	−0.36	0.115

## Data Availability

Data supporting the reported results can be provided upon reasonable request by the corresponding author.

## Abbreviations

BMI	Body mass index
CI	Confidence interval
DASH	Disabilities of the Arm, Shoulder, and Hand
FTSG	Full thickness skin grafting
IQR	Interquartile ranges
PDS	Polyester poly(p-dioxanone) suture
POSAS	The Patient and Observer Scar Assessment Scale
RFFF	Radial forearm free flap
SBSES	The Stony Brook Scar Evaluation Scale
